# Deep Sequencing of Foot-and-Mouth Disease Virus Reveals RNA Sequences Involved in Genome Packaging

**DOI:** 10.1128/JVI.01159-17

**Published:** 2017-12-14

**Authors:** Grace Logan, Joseph Newman, Caroline F. Wright, Lidia Lasecka-Dykes, Daniel T. Haydon, Eleanor M. Cottam, Tobias J. Tuthill

**Affiliations:** a The Pirbright Institute, Pirbright, Surrey, United Kingdom; b Institute of Biodiversity, Animal Health and Comparative Medicine, College of Medical, Veterinary and Life Sciences, University of Glasgow, Glasgow, United Kingdom; University of Texas Southwestern Medical Center

**Keywords:** RNA packaging, nonenveloped, picornavirus, virus assembly

## Abstract

Nonenveloped viruses protect their genomes by packaging them into an outer shell or capsid of virus-encoded proteins. Packaging and capsid assembly in RNA viruses can involve interactions between capsid proteins and secondary structures in the viral genome, as exemplified by the RNA bacteriophage MS2 and as proposed for other RNA viruses of plants, animals, and human. In the picornavirus family of nonenveloped RNA viruses, the requirements for genome packaging remain poorly understood. Here, we show a novel and simple approach to identify predicted RNA secondary structures involved in genome packaging in the picornavirus foot-and-mouth disease virus (FMDV). By interrogating deep sequencing data generated from both packaged and unpackaged populations of RNA, we have determined multiple regions of the genome with constrained variation in the packaged population. Predicted secondary structures of these regions revealed stem-loops with conservation of structure and a common motif at the loop. Disruption of these features resulted in attenuation of virus growth in cell culture due to a reduction in assembly of mature virions. This study provides evidence for the involvement of predicted RNA structures in picornavirus packaging and offers a readily transferable methodology for identifying packaging requirements in many other viruses.

**IMPORTANCE** In order to transmit their genetic material to a new host, nonenveloped viruses must protect their genomes by packaging them into an outer shell or capsid of virus-encoded proteins. For many nonenveloped RNA viruses the requirements for this critical part of the viral life cycle remains poorly understood. We have identified RNA sequences involved in genome packaging of the picornavirus foot-and-mouth disease virus. This virus causes an economically devastating disease of livestock affecting both the developed and developing world. The experimental methods developed to carry out this work are novel, simple, and transferable to the study of packaging signals in other RNA viruses. Improved understanding of RNA packaging may lead to novel vaccine approaches or targets for antiviral drugs with broad-spectrum activity.

## INTRODUCTION

The Picornaviridae is a large virus family containing over 30 genera and includes viruses of medical and veterinary significance, such as poliovirus, human rhinovirus, and foot-and-mouth disease virus (FMDV). Picornaviruses have a genome of single-stranded positive-sense RNA usually of between approximately 7,200 and 8,500 nucleotides within a nonenveloped capsid approximately 30 nm in diameter.

Virus assembly involves the multimerization of five copies of a capsid precursor to form a pentameric capsid subunit, followed by assembly of 12 of these pentamers into intact capsids. During this process, a molecule of correctly folded genomic RNA must be encapsidated or packaged into the assembling capsid. Only newly replicated poliovirus RNA is packaged (1), suggesting a link between RNA replication and capsid assembly. For many picornaviruses, a proportion of capsids in the infected cell assemble without encapsidating the viral genome, and such empty capsids also can be readily assembled in recombinant expression systems. The exclusion of nonviral RNA from such particles suggests that packaging is specific for viral RNA, but the interactions driving packaging and conferring specificity remain poorly understood. In other nonenveloped RNA viruses, packaging is known to involve interactions between capsid proteins and secondary structures in the viral genome (packaging signals), as exemplified by the bacteriophage MS2 (2) and plant viruses (3, 4). A packaging signal comprising an RNA stem-loop was previously identified for one virus in the picornavirus family, Aichi virus (5, 6). In contrast, no such RNA packaging signal has been reported yet for the most well-studied virus in the family, poliovirus, and interaction between genome and capsid required for packaging has instead been shown to be mediated via an additional viral protein, nonstructural protein 2C (7–9). Recently, however, contacts between capsid and RNA have been visualized in the virion structures of picornaviruses in the parechovirus genus (10–12), and multiple structured RNA motifs which interact with the capsid of human parechovirus have been confirmed as packaging signals required for virus assembly (13).

Picornavirus genomes are replicated by a virally encoded RNA-dependent RNA polymerase which lacks proof-reading function. Replication therefore introduces errors, resulting in viral genomes existing as a population or swarm of closely related RNA molecules (14). In addition, packaging of picornavirus genomes is generally accepted to be incomplete, such that only a proportion of genomes in an infected cell are packaged into viral capsids. We therefore hypothesized that within the total population of viral genomes in the cell, some genomes would contain substitutions that were detrimental to packaging. These genomes would be packaged less efficiently and would be underrepresented so that sequence diversity would be constrained at these positions in the packaged population of genomes.

## RESULTS AND DISCUSSION

**FIG 1:**
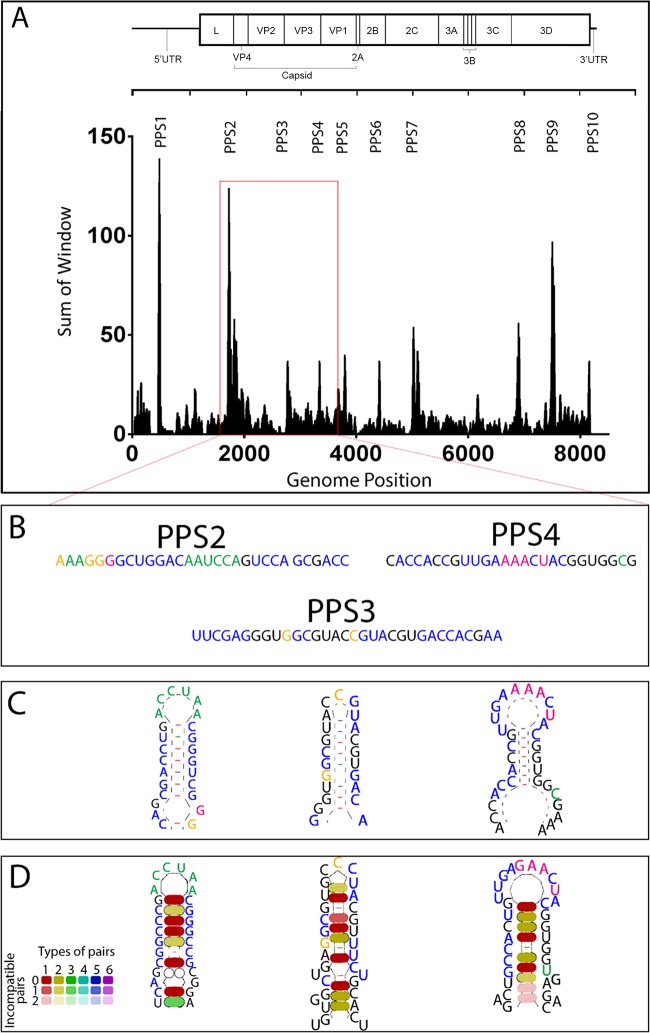
Specific regions of the genome are more conserved in the packaged population and are predicted to form stem-loop structures. (A) Graph showing a sliding window analysis to highlight the differences in Shannon's entropy between the total RNA population and the packaged RNA population. A window of 19 nt was slid across the genome, and each nucleotide was scored according to the change in entropy at that position. Nucleotides in the top 50 positions (approximately 0.6% of nucleotides) for the highest change in entropy across the genome scored 12 points, positions 51 to 100 scored 6 points, positions 101 to 150 scored 3 points, and all other positions with constrained entropy scored 1 point. The sum of these scores within the 19-nt window is shown (*y* axis) against the nucleotide position in the center of the window (*x* axis). Regions with the highest peaks are termed putative packaging signals 1 to 10 (PPS1 to PPS10). Regions that were within 50 nt of one another were combined and analyzed as one region of interest. The red box indicates the area containing PPS2, PPS3, and PPS4. (B) The nucleotide sequences of PPS2, PPS3, and PPS4 are shown. Nucleotides are color coded according to the change in entropy at that position between the packaged and total populations. Green indicates nucleotides in the top 50 positions (approximately 0.6% of nucleotides) showing the highest change in entropy score across the entire genome, orange represents positions 51 to 100, pink represents 101 to 150, blue represents the remainder of positions with constrained entropy, and black represents positions with no constraint. (C) RNA secondary structures predicted by Mfold for the nucleotide sequences shown in panel B. (D) Conservation of base pairing to maintain predicted secondary structures using the LocaRNA tool for multiple alignment of 59 diverse isolates of FMDV type O. GenBank accession numbers for the FMDV sequences are AY593813, AY593811, AY593815, AY593817, AY593819, AY593821–AY593837, AY686687, EF552688–EF552697, EF614457, EU214601, FJ175662–FJ175666, FJ542365–FJ542371, GU384682, GU384683, HM008917, HM229661, HQ009509, HQ412603, HQ632769–HQ632772, JN998085, and JN998086. The overall consensus sequence and predicted structure of these sequences is shown. An additional color scheme is used to represent the number of different compatible base-pairing combinations present at each position in the predicted stem across all 59 viruses analyzed (single combination of nucleotides, red; 2 different combinations, yellow; 3 combinations, green). The shading intensity indicates the number of incompatible pairs (no incompatible pairs, intense color; 1 incompatible pair, 50% shading; 2 incompatible pairs, light shading; >2 incompatible pairs, no color). The predominance of intense red and yellow shading indicates high levels of conservation of base-pairing across the panel of viruses analyzed.

FMDV (type O1K)-infected cell cultures were lysed to generate a sample containing both packaged and unpackaged genomes, and this was designated the total population of viral genomes. Virus particles were also purified from a portion of this sample by ultracentrifugation through sucrose gradients to generate a sample containing only packaged genomes, designated the packaged population. The populations of genomes in these samples were analyzed by deep sequencing. For both total and packaged populations, the variability at each nucleotide position in the genome was calculated using Shannon's entropy (15). For each position in the genome, the entropy value for the packaged population was subtracted from the entropy value for the total population. Therefore, positive values in this analysis represent positions in the genome where variation is constrained in the packaged population relative to the total population. This highlighted several clusters of such nucleotides spread along the length of the genome (Fig. 1A). In recognition of the hypothesis that these sequences were involved in packaging, the 10 most prominent of these clusters of constraint were designated putative packaging signals (PPS1 to PPS10).

Consensus sequences spanning each of these PPS were analyzed for predicted secondary structure using Mfold (16, 17). This revealed that each of these 10 regions contained predicted stem-loop structures featuring base pairing to form a stem of between 11 and 28 nucleotides. This presented an unpaired loop of between 4 and 9 nucleotides containing, on average, 74% adenine or cytosine. The secondary structure of these regions, as predicted by Mfold, is outlined in Table 1. Three sequences containing such secondary structures and referred to as PPS2, PPS3, and PPS4 were targeted for further analysis. These sites were used because PPS2 showed a very clear signal in the entropy analysis and because the sequence containing the three sites was conveniently flanked by restriction enzyme sites which would facilitate the insertion of modified sequences into full-genome cDNA plasmids.

**TABLE 1:**
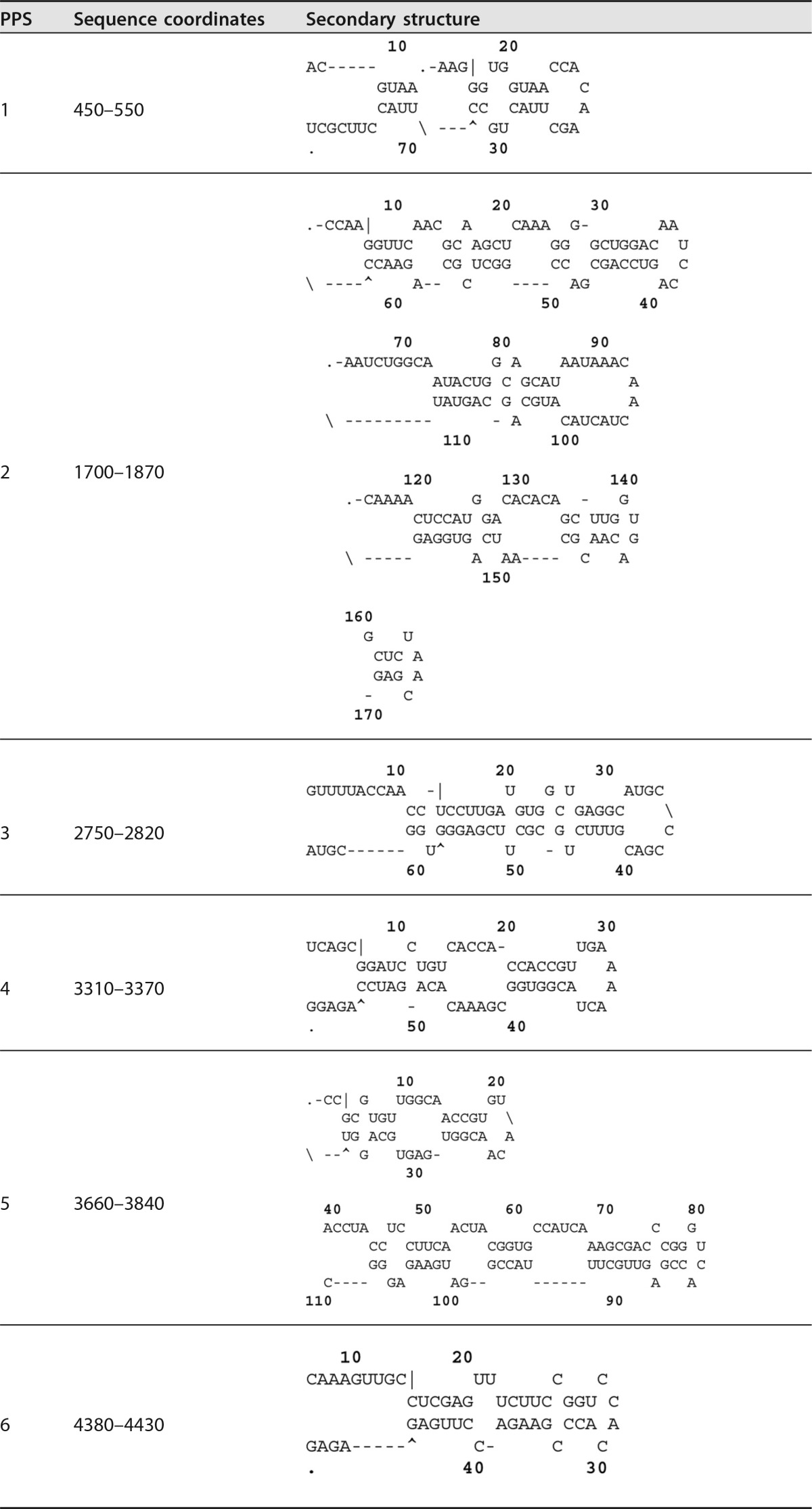

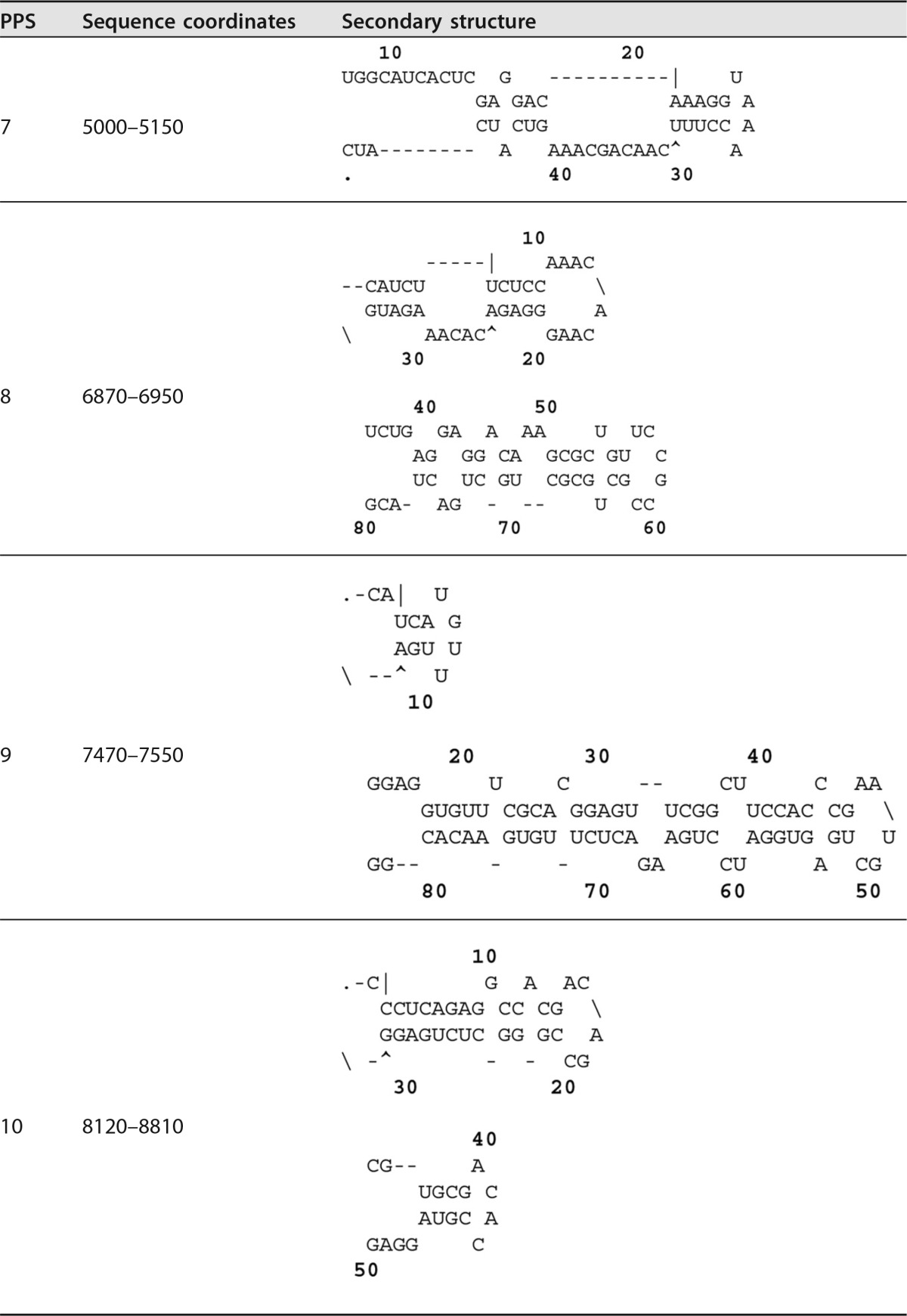
Location and predicted RNA secondary structure of each of the putative packaging signals^*a*^

a Sequence coordinates of each region are shown in reference to the FMDV O1K genome, and secondary structures are shown as predicted by Mfold.

In order to highlight positions within these sequences with the highest constraint in the packaged population, individual nucleotides were color coded according to the values for constraint in sequence entropy as described above (Fig. 1B). This showed that for PPS2 and PPS4, the maximum constraint in sequence entropy coincided with the unpaired sequence at the top of the predicted stem loops (Fig. 1C). The sequences spanning these predicted structures were compared with the equivalent sequences in 59 diverse type O virus isolates using LocaRNA (18–20) to identify conservation of predicted RNA structures. This showed that despite variation in primary sequence between isolates, there was conservation of the base pairing required to maintain the stem-loop structures (Fig. 1D). The loop sequences predicted to be at the ends of these stem-loops were also highly conserved between isolates. The loop of PPS2 contains 6 nucleotides (nt) (AAUCCA). The first, third, fourth, and sixth nucleotides are 100% conserved between the 59 isolates compared in the LocaRNA analysis. Nucleotide 2 is 97% conserved (2/59 genomes have a G instead of an A), while nucleotide 5 is 98% conserved (1/59 isolates has a U instead of a C). The loop of PPS3 contains 3 nt (CCC). The latter two nucleotides are 100% conserved in the 59 isolates compared. The first position is 91% conserved, with 5/59 isolates showing a U in place of a C at this position. PPS4 has a loop containing 8 nt (UGAGAACU). Nucleotides 2, 3, 5, and 8 are 100% conserved. Nucleotides 1 and 7 are 93% conserved (with 4/59 isolates having a C instead of a U and a U instead of a C, respectively). Nucleotide 4 is 88% conserved, with 7 isolates having an A at this position instead of a G, and nucleotide 6 is 98% conserved, with 1 isolate with a G instead of an A at this position. This suggested that the predicted secondary structures tentatively identified by deep sequencing of samples derived from a single virus isolate were maintained in all viruses within this serotype.

**FIG 2:**
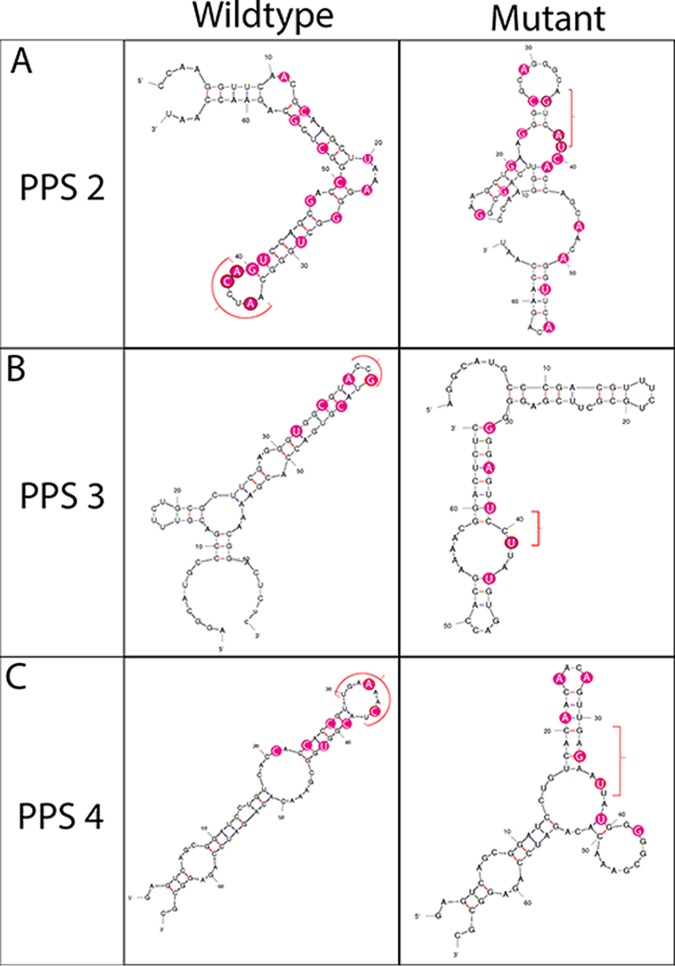
Silent mutagenesis disrupts predicted secondary structures. RNA structures predicted by Mfold are shown in wild-type (left column) and mutated (right column) sequences spanning PPS2 (A), PPS3 (B), and PPS4 (C). Nucleotides identified with a red bracket represent positions at the top of the stem-loop structures in the wild-type sequences and their altered positions in the disrupted structures after mutagenesis. Nucleotides highlighted in pink represent positions of mutations. Wild-type and mutated sequences are also shown in Table 2.

Silent mutagenesis of PPS2, PPS3, and PPS4 was designed to disrupt or destabilize the secondary structures predicted by Mfold in these regions without altering the encoded amino acid sequence (Fig. 2). A single fragment of synthetic DNA containing the mutated sequences was introduced into an infectious cDNA plasmid and mutant, and wild-type viruses were recovered by transfecting BHK-21 cells with *in vitro*-transcribed RNA. Recovered viruses were passaged twice on the same cell line and harvested at appearance of complete cytopathic effect (CPE) to generate virus stocks for the following analysis.

**FIG 3:**
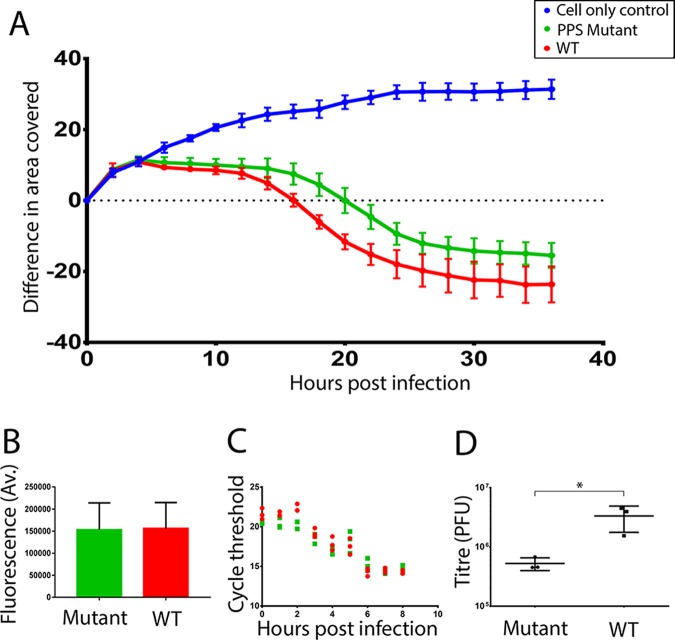
Mutations in putative packaging signals reduce rate of virus spread and infectious titer without affecting replication and translation of viral RNA. (A) Virus growth from low-MOI infection shown as development of CPE and resultant reduction in area covered by cells (*y* axis) against time postinfection (*x* axis). Wild-type virus, red; mutated virus, green; uninfected cells, blue. The mutated virus (green line) spreads through the culture more slowly than the wild type (red line). Data shown are the means from 7 replicates ± standard deviations (SD) and are representative of multiple separate experiments. (B) Similar immunofluorescence signals for nonstructural protein 3A in cells 4 h postinfection with wild type (WT; red bar) and mutant (green bar) virus. Data shown are mean fluorescence per cell across seven separate wells (and from at least 500 infected cells/well) ± SD and are representative of multiple separate experiments. (C) Similar levels of viral RNA in cells at time points indicated postinfection with wild-type (red) and mutant (green) virus. Data are shown as cycle threshold (*C_T_*) number from RT-qPCR. Three technical replicates are shown. Data are representative of multiple separate experiments. (D) Comparison of infectious virus titer as PFU at 5 h postinfection of cells with mutant and wild-type (WT) virus. Infections were initiated at equal MOI. Titer of three technical replicates is shown on a log scale with SD. *P* = 0.039 by two-sample *t* test. Data are representative of multiple separate experiments.

In order to quantitate the rate of spread in a monolayer of mutant and wild-type viruses in culture, a cell-killing assay was developed using an imaging plate reader. Using this assay, experiments initiated at equal multiplicities of infection (MOI) showed that the rate of spread through the culture of the mutant virus was slower than that of the wild type (Fig. 3A). In these experiments, detection of nonstructural protein 3A by immunofluorescence was used to confirm that mutant and wild-type viruses infected the same number of cells in the first round of infection. Quantitation of the immunofluorescence signals showed that both viruses also produced comparable levels of viral proteins in cells in the first round of infection (Fig. 3B). Similarly, reverse transcription-quantitative PCR (RT-qPCR) was used to show that both viruses produced equivalent levels of viral RNA during the first round of infection (Fig. 3C). Despite this, the titer of infectious virus produced by the mutant in a single round of infection (5 h postinfection) was approximately 1 log less than that of the wild type (Fig. 3D). These findings suggested that viral RNA replication and production of viral proteins by the mutant virus was not impaired within cells. However, the reduced viral titer and slow spread through the culture suggested a partial defect in the assembly of infectious particles by the mutated virus. It was noticed that the plaques generated by the mutant virus were marginally less clear in appearance relative to the wild type but were not significantly different in size, suggesting that the reduced production of infectious particles by the mutant virus was not sufficient to significantly slow the cell-to-cell spread required for plaque formation.

**FIG 4:**
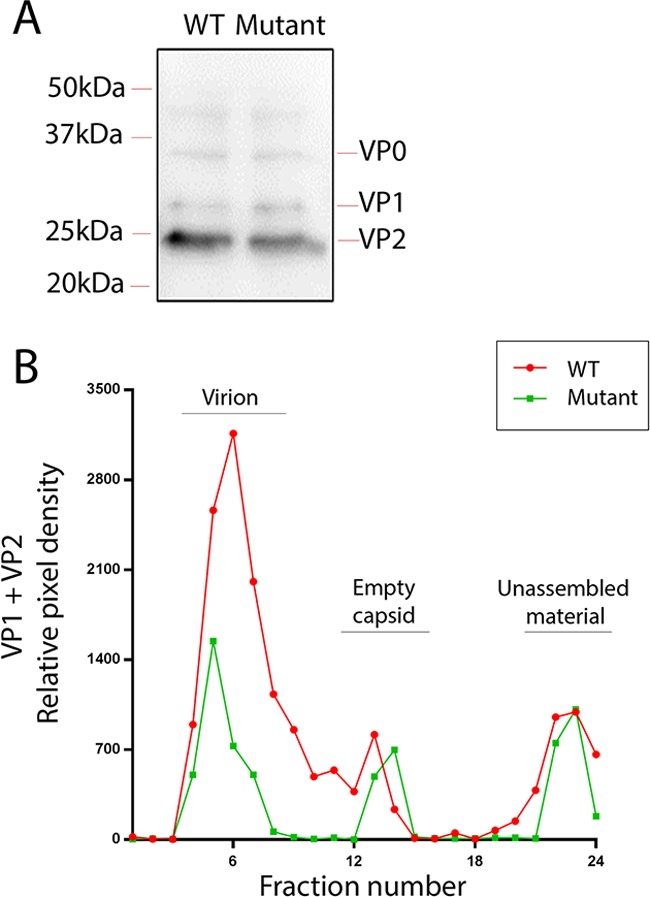
Mutations in putative packaging signals reduce the assembly of RNA-containing virions. (A) Immunoblots using anti-FMDV sera show equivalent signal for total viral capsid material in cells infected with wild-type (WT) or mutant virus. (B) Separation of RNA-containing virions, empty capsids, and unassembled capsid precursors by sedimentation of capsid material derived from cells infected with WT (red) or mutant (green) virus. Capsid material in gradient fractions was detected by immunoblotting and quantitated by densitometry. The expected positions on gradients of virions, empty capsids, and unassembled material are indicated. The data shown are representative of multiple experiments.

The assembly of virus particles by the mutant and wild-type viruses was therefore compared. Cell cultures were infected with mutant and wild-type viruses and allowed to reach complete CPE, and lysates were prepared by freeze-thawing. The total amount of capsid material in the cell lysates was detected by SDS-PAGE and Western blotting using an anticapsid polyclonal sera. The signal for capsid material was comparable for both viruses (Fig. 4A), showing that the amount of total capsid material in the lysates was similar. This was in agreement with the immunofluorescence data described above. In order to differentiate virus, empty capsids, and unassembled capsid precursors, the lysates were subjected to ultracentrifugation through sucrose density gradients that would separate these different forms of the capsid according to their rate of sedimentation. Gradients were fractionated and capsid material detected by Western blotting as described above, and Western blot signals were quantitated by densitometry to allow gradient profiles to be displayed as line graphs. In the wild-type lysate a predominant peak in the expected position for virus particles was observed (Fig. 4B). In the mutant virus lysate this peak was clearly reduced in size (Fig. 4B), suggesting that the mutant virus was forming fewer mature virus particles than the wild type. This was consistent with the mutant virus producing a lower infectious titer and was likely due to the introduced mutations causing a partial defect in packaging of genomic RNA into the capsid. We had anticipated that if fewer mature virus particles were formed, a corresponding increase in the signal for empties or precursors might be observed, but this was not the case. This could be explained by the accumulation of unassembled precursors becoming insoluble such that they would no longer be represented in the sedimentation analysis of soluble proteins.

Recent structural studies of picornaviruses in the parechovirus genus have identified contacts between the viral genome and the inside surface of the capsid (10–13). We propose that the predicted secondary structures identified in the current study are the equivalent positions where the FMDV RNA interact with capsid precursors in order for the genome to be correctly folded and packaged into the assembling capsid. Studies with poliovirus have shown that RNA packaging requires an interaction between the capsid and viral nonstructural protein 2C (7, 8). This has not been investigated in the current study, and the data presented here do not exclude a similar involvement of 2C in FMDV packaging.

The number of PPS (10) initially highlighted in this study was close to 12, suggesting a mechanism for packaging where 12 sites on the genome each interact with one of the 12 pentamers which assemble together to form the intact capsid. However, only 3 of these sites were analyzed in more detail as described above. Therefore, this study provides evidence for the likely involvement of interactions between capsid and genome in the packaging of FMDV but is not able to specify the exact number of sites in the genome that might be involved. The parechovirus structures described above (10–12) and the recent identification of parechovirus packaging signals (13) suggest that interactions between the genome and capsid occur at 60 positions (equivalent to one interaction per capsid monomer), not at just 12 positions (one on each of the 12 pentamers).

In summary, this study has used deep sequencing to identify predicted secondary structures or putative packaging signals in the genome of FMDV which are required for efficient assembly of infectious, RNA-containing virus particles. This approach offers a readily transferable methodology for investigating packaging requirements in many other viruses.

## MATERIALS AND METHODS

### Cells, viruses, and antibodies.

The baby hamster kidney cell line (BHK-21; Pirbright Central Services Unit) was cultivated in Glasgow's modified Eagle's medium supplemented with 10% fetal calf serum, 5% tryptose phosphate broth, 20 mM glutamine, penicillin (100 SI units/ml), and streptomycin (100 μg/ml). FMDV strain O1 Kaufbeuren was derived from an infectious copy plasmid, pT7S3 (21). Monoclonal antibody 2C2, which recognizes FMDV 3A, was a previous gift from E. Brocchi (IZSLER, Brescia, Italy) to T. Jackson (Pirbright).

### Virus purification.

BHK-21 cells were infected with FMDV and freeze-thawed at the point of complete CPE, and the resulting lysate was clarified by low-speed centrifugation. The supernatant was precipitated with saturated ammonium sulfate, pelleted at 4,800 × *g* at 4°C for 1 h, and resuspended in phosphate-buffered saline (PBS) (pH 7.4) containing 1% IGEPAL CA-630. Virus was pelleted through a 30% sucrose cushion at 100,000 × *g* for 2.5 h, resuspended as described before, and purified by sedimentation through a 15 to 45% sucrose gradient by centrifugation at 100,000 × *g* for 2.5 h. Purified virus was located by measuring the 260-nm absorbance of gradient fractions.

### Next-generation sequencing.

RNA was extracted from samples of clarified infected cell lysate or purified virus using TRIzol and prepared for next-generation sequencing as described previously (22). Briefly, reverse transcription used both random hexamers and FMDV-specific primers. Subsequent DNase treatment and cleanup was followed by second-strand synthesis before library preparation using Nextera XT reagents (Illumina) and sequencing on the MiSeq v2 (Illumina). Although originally described as a consensus-level sequencing methodology, depth of coverage was such that deep sequencing analysis also could be carried out. Bioinformatics analysis of the data was completed using the pipeline previously described (22). The windowed adaptive quality trimming tool Sickle was used to trim reads using Q30 as a cutoff. A *de novo* reference sequence was created with Velvet (using Velvet optimizer), and reads subsequently were aligned to this reference using Bowtie2. Shannon's entropy was calculated using R.

### Mutagenesis and virus recovery.

For each of PPS2, PPS3, and PPS4, a sequence was designed which disrupted the predicted secondary structure in that region without altering amino acid coding (Table 2 and Fig. 2). A single fragment of synthetic DNA spanning the region containing all three modified sequences was synthesized (GeneArt; ThermoFisher Scientific) and transferred into a full-length DNA copy of the FMDV genome in the plasmid pT7S3 using standard molecular biology approaches and the restriction sites XmaI and AflII. The sequence of the modified plasmid was confirmed by conventional sequencing. The modified plasmid (pT7S3_Mutant) and parental wild-type plasmid (pT7S3_WT) were used as templates to generate T7 RNA transcripts and mutant and WT viruses recovered by transfection of RNA into BHK-21 cells. Recovered viruses were passaged twice in BHK-21 cells to produce working stocks for subsequent experiments.

**TABLE 2:** Point mutations made in PPS mutant infectious cDNA plasmid^*a*^

PPS	Sequence (5′–3′)
PPS2 WT	CC AAG GUU CA**A** CG**C** AAG CU**U** AA**A** GG**G** GC**U** GGG CA**A** UC**C AGU** CCA GC**G** AC**C** GG**C** UC**G** CAG AAC CAA U
PPS2 Mut	CC AAG GCC CA**G** CG**G** AAG CU**G** AA**G** GG**C** GC**A** GGG CA**G** UC**A UCA** CCA GC**A** AC**A** GG**U** UC**A** CAG AAC CAA U
PPS3 WT	AG GCA UGC CCG ACG UUU CUG CGC UUC GAG GG**U** GG**C** GU**A** CC**G** UA**C** GUG ACC ACG AAA ACG GAC UCU C
PPS3 Mut	AG GCA UGC CCG ACG UUU CUG CGC UUC GAG GG**G** GG**A** GU**U** CC**U** UA**U** GUG ACC ACG AAA ACG GAC UCU C
PPS4 WT	GAG UCA GCG GAU CCU GUC AC**C** AC**C** AC**C** GUU GA**A** AA**C** UA**C** GG**U** GGC GAA ACA CAG AUC CAG AGG CGC
PPS4 Mut	GAG UCA GCG GAU CCU GUC AC**A** AC**A** AC**A** GUU GA**G** AA**U** UA**U** GG**G** GGC GAA ACA CAG AUC CAG AGG CGC

a Boldface type indicates nucleotides where mutations were introduced in the mutant (Mut) relative to the wild type (WT).

### Cell killing assay.

Subconfluent BHK-21 cells in 96-well plates were infected with WT or mutant viruses at low MOI (0.2), resulting in conditions where spread through the culture and appearance of CPE and cell death occurred over approximately 2 days. The MOI for WT and mutant viruses was confirmed as equal by immunofluorescence. The appearance of CPE was recorded by capturing transmitted light images of each well every hour postinfection for 36 h using an imaging plate reader (SpectraMax MiniMax 300; Molecular Devices). The surface area covered by cells was automatically calculated for every image using image analysis software (SoftMax Pro; Molecular Devices). As cells died, the surface area covered became less, allowing quantitation of the rate of virus growth through the culture. Data were visualized using GraphPad prism.

### Immunofluorescence.

Cells were fixed 4 h postinfection with 4% formaldehyde in PBS, permeabilized with 0.1% Triton X-100, and blocked with 0.5% bovine serum albumin (BSA) in PBS. The fixed monolayers were serially incubated with monoclonal antibody 2C2 (specific for FMDV nonstructural protein 3A) at 1:1,000 in block buffer, Alexa Fluor 488-conjugated anti-mouse secondary antibody (Invitrogen) at 1:200 dilution in blocking buffer, and the nuclear counterstain TO-PRO-3 (ThermoFisher Scientific). Immunofluorescence images were captured using an imaging plate reader (SpectraMax MiniMax 300; Molecular Devices). Quantitation of the total number of cells based on TO-PRO-3 fluorescence, number of infected cells based on Alexa Fluor 488 fluorescence, and intensity of signal for each cell was automated by image analysis software (SoftMax Pro; Molecular Devices). Data were visualized using Prism 7 (GraphPad).

### Immunoblotting to detect FMDV capsid proteins.

Standard methods were used for the separation of samples by 12% SDS-PAGE and transfer of proteins onto 0.45-μm-pore-size nitrocellulose membranes (GE Healthcare). Membranes were blocked in 1% fish skin gelatin (Sigma) in 20 mM Tris, pH 7.6, 150 mM NaCl, 0.1% Tween 20 before being incubated with guinea pig anti-FMDV O1M sera (Pirbright World Reference Laboratory) at 1:1,000 dilution in blocking buffer overnight at 4°C. Membranes then were incubated with rabbit anti-guinea pig horseradish peroxidase-conjugated antibodies (Dako) at 1:3,000 dilution in blocking buffer for 1 h at room temperature. The location of proteins was revealed using a chemiluminescence substrate (SuperSignal West Pico; ThermoFisher Scientific) and a gel documentation system (G:Box Chemi xx6; Syngene). Where required, the intensity of the signal for FMDV capsid proteins was quantitated by densitometry using NIH ImageJ (v1.50). Various exposures were collected to ensure band intensity was within the linear range of the equipment. Data showing relative signal intensity were presented using Prism 7 (GraphPad).

### Sucrose density gradients to differentiate virus capsids and unassembled precursors.

Continuous gradients of 10 to 30% sucrose in PBS were prepared in 5-ml ultracentrifuge tubes using a gradient master (Biocomp). The top 200 μl was removed and replaced with 200 μl of sample, and gradients were centrifuged in an SW55 rotor (Beckman) at 286,794 × *g* for 44 min at 8°C. Gradients were fractionated into 24 equal 240-μl fractions using a repeating microdispenser (Acura 865; Socorex). FMDV capsid components in gradient fractions were detected by immunoblotting as described above.
